# *Drosophila* ClC-c Is a Homolog of Human CLC-5 and a New Model for Dent Disease Type 1

**DOI:** 10.34067/KID.0000000000000352

**Published:** 2024-01-18

**Authors:** Carmen J. Reynolds, Christopher M. Gillen, Richard Burke, Yula Tsering, Emi Loucks, Sebastian Judd-Mole, Julian A.T. Dow, Michael F. Romero

**Affiliations:** 1 Physiology & Biomedical Engineering, Mayo Clinic College of Medicine & Science, Rochester, Minnesota; 2 Department of Biology, Kenyon College, Gambier, Ohio; 3 School of Biological Sciences, Monash University, Melbourne, Victoria, Australia; 4 University of Minnesota-Rochester, Rochester, Minnesota; 5 School of Molecular Biosciences, College of Medical Veterinary and Life Sciences, University of Glasgow, Glasgow, United Kingdom; 6 Nephrology and Hypertension, Mayo Clinic College of Medicine & Science, Rochester, Minnesota

**Keywords:** genetic renal disease

## Abstract

**Key Points:**

- *Drosophila* can be a model for Dent Disease type 1.
- *Drosophila* Clc-C mutations function similar to human CLC-5 Dent 1 mutations.

**Background:**

*Drosophila* serve as exceptional alternative models for *in vivo* and *ex vivo* research and may provide an avenue for in-depth investigation for human ClC-5 and Dent disease type 1 (DD1). The *Drosophila* ClC-c (CG5284) has sequence homology with human ClC-5 and is hypothesized to encompass similar functional and phenotypical roles with ClC-5 and variants that cause DD1.

**Methods:**

Ion transport function and activity of *Drosophila* ClC-c and homologous DD1 variants were assessed by voltage clamp electrophysiology. Membrane localization was demonstrated in *Drosophila* expressing a GFP-labeled construct of ClC-c. Genetic expression of an RNAi against ClC-c mRNA was used to generate a knockdown fly that serves as a DD1 disease model. Tubule secretion of cations and protein were assessed, as well as the crystal formation in the Malpighian tubules.

**Results:**

Voltage clamp experiments demonstrate that ClC-c is voltage-gated with Cl^−^-dependent and pH-sensitive currents. Inclusion of homologous DD1 mutations pathogenic variants (S393L, R494W, and Q777X) impairs ClC-c ion transport activity. *In vivo* expression of ClC-c-eGFP in Malpighian tubules reveals that the membrane transporter localizes to the apical membrane and nearby cytosolic regions. RNAi knockdown of ClC-c (48% decreased mRNA expression) causes increased secretion of both urinary protein and Ca^2+^ and increased occurrence of spontaneous tubule crystals.

**Conclusions:**

*Drosophila* ClC-c shows orthologous function and localization to human ClC-5. Thus, *Drosophila* and ClC-c regulation may be useful for future investigations of Cl^−^ transport, Ca^2+^ homeostasis, and urinary protein loss in DD1.

## Visual Abstract

**Figure:**
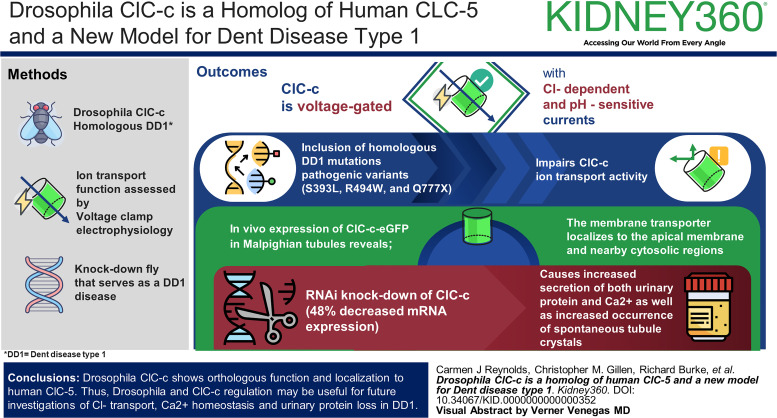

## Introduction

The human voltage-gated 2Cl^−^/H^+^ exchange transporter, ClC-5, is an epithelial membrane exchanger that facilitates protein and Ca^2+^ absorption in the kidney.^1–6^ The physiological role of ClC-5 is portrayed by the clinical characteristics of patients with Dent disease type 1 (DD1) who have a disease-causing variant in the X-chromosome gene, *CLCN5*. Affected individuals with DD1, primarily men, exhibit both low molecular weight proteinuria (LMWP) and hypercalciuria and are particularly prone to nephrocalcinosis, nephrolithiasis, and chronic kidney disease or renal failure in some patients.^7–11^ The notion that an impaired Cl^−^ transporter can cause Ca^2+^ dysregulation suggests an intricate relationship among these ions that remains unresolved because of the limitations of experimental models. Given the limited treatment options available for patients with DD1, achieving a better understanding of the underlying mechanisms of disease could lead to much-needed therapeutic targets.

Current model systems to determine the mechanistic actions of ClC-5 are limited to cultured cells and knockout mice. Much of ClC-5's structural and functional characteristics have been deciphered *in vitro* from cells expressing ClC-5^5,12–14^ or *via* homologous endogenous Cl^−^ transporters.^15–21^ Cell culture, unfortunately, cannot appropriately recapitulate altered Ca^2+^ metabolism. Mouse models have been generated by knockout of ClC-5, but these mice do not form kidney stones and experience minimal deleterious effects outside of the kidney.^22–24^ To gain a better understanding on the dynamics of Ca^2+^ dysregulation, we need to use a model organism that demonstrates the Ca^2+^ dysregulation and allows for whole organism experimentation.

Despite an evolutionary distance from mammals, *Drosophila melanogaster* serve as a useful model for studying fundamentals of biological processes (*e*.*g*., olfaction, innate immunity, circadian rhythms).^25–27^ Insects use Malpighian tubules (MTs) to secrete solutes from the hemolymph and produce urine similarly to renal epithelia. Circulating hemolymph is filtered by nephrocytes equipped with slit diaphragm structures and proteins akin to mammalian podocytes.^28,29^ Solutes are removed by principal cells of the MT that are rich with luminal microvilli while fluid homeostasis is maintained by the MT stellate cells.^30^ *Drosophila* in particular have been used to study renal epithelial ion and water secretion, as well as models for several genetic kidney diseases.^31^ Urine secretions from the MT can be quantified per individual tubule if sensitive analytical equipment is available.^31^ Within MTs, crystals can form that are comparable with intratubular microlithiasis occurring in human kidneys. These crystals appear as bright white structures under polarized light microscopy and can be measured by image analysis.^32–34^ Not only are secretions and crystal formation by the MT quantifiable but the rapid fecundity of *Drosophila* lends well to genetic manipulations with the GAL4/UAS-directed expression or CRISPR/Cas9 editing methods.

Here, we introduce a *Drosophila* model for studying the mechanistic function of this Cl^−^ transporter. We evaluate biophysical properties of ClC-c as a homolog to ClC-5 and assess mutations in ClC-c that correspond to select DD1 disease variants identified by the Rare Kidney Stone Consortium, Dent Disease Registry, including S244L, R345W, and Q629X.^35–37^ In addition, we characterize the effect of ClC-c RNAi knockdown in *Drosophila* in replicating DD1 features.

## Methods

### Molecular Biology

*Drosophila* ClC-c was subcloned from MTs of *w*^*1118*^ flies into pGEMHE *Xenopus* oocyte and into pUASTattB:eGFP *Drosophila* expression plasmids using primers described (Supplemental Table 1). Mutations were incorporated by site-directed mutagenesis for S393L, R494W, and Q777X into pGEMHE:ClC-c. The pGEMHE:ClC-c construct was amplified with respective mutagenesis primers by Phusion Hot Start II DNA Polymerase PCR (30 cycles of 98°C [2s]: 72°C [120s]; Thermo Fisher Scientific) and then treated with Dpn1 (New England Biolabs) at 37°C for 1 hour. PCR products were purified, transfected into confluent bacteria, and grown with ampicillin. Plasmids were isolated from bacteria by Qiagen Maxi-prep, and the concentrations were determined by nanodrop spectrometer. Mutations were confirmed by Sanger sequencing (Genewiz).

### cRNA Synthesis

DNA constructs cloned into the pGEMHE expression vector were linearized by Not1 or Nhe1 (New England Biolabs, Ipswich, MA) restriction digest overnight at 37°C. cRNA was synthesized from the linear vector using the Invitrogen mMessage Machine T7 Transcription Kit (Thermo Fisher Scientific, Waltham, MA).

### *Xenopus laevis* Oocytes

Frogs were maintained in accordance with and approved by the Institutional Animal Care and Use Committee of the Mayo Clinic College of Medicine & Science. Oocytes were collected by surgical laparotomy and follicular membranes digested as described previously.^38^ Cells were maintained in Leibovitz's 15 Media (Thermo Fischer, Waltham, MA) with 2.5% penicillin/streptomycin (Gibco, Thermo Fischer, Waltham, MA) and 1 mM HEPES at 16°C. One day after collection, oocytes were injected with 10 ng cRNA (in 50 nl H_2_O) or vehicle and then experiments were performed 3–6 days after injection.

### Electrophysiology

Two-electrode voltage clamp experiments were performed on oocytes with an oocyte clamp amplifier (OC-725C, Warner Instruments, Hamden CT) using Heka software (Wiesenstrasse, Germany) as previously described.^36,37^ Briefly, electrodes filled with 3M KCl micropipette solution were inserted into oocytes submerged in ND96 solution (96 mM NaCl, 2 mM KCl, 1.8 mM CaCl_2_ 1 mM MgCl_2_, 5 mM HEPES, pH 7.5) at room temperature. Membrane voltage was maintained at −60 mV, and currents were recorded for 75 ms pulses from −80 mV to +80 mV in +20 mV increments. Experiments were repeated with alternate ND96 solutions at pH 6.0, pH 8.5 (pH adjusted by NaOH), or 0 Cl^−^ (96 mM Na-gluconate, 2 mM K-gluconate, 1.8 mM Ca-gluconate 1 mM Mg-gluconate, 5 mM HEPES, pH 7.5). Conductance was calculated as <conductance = current/voltage = amps/volts = Siemens> as current/voltage, or the average was determined by slope calculation of a best-fit linear equation.

### Drosophila

*D*. *melanogaster* stock lines and breeding crosses were maintained at 20°C and 22°C, respectively, on a 12:12 hours light:dark cycle and fed standard diet, *ad libitum*. Experimental lines were generated by breeding male flies that express the GAL4 driver in MT principal cells [*w*^*−*^;*Uro*-GAL4/*Uro*-GAL4;^+/+^ and *w*^−^;^+/+^;*c507*-GAL4/*c507*-GAL4 (Dow Lab)] with virgin female flies with UAS promoted constructs [*w*^*−*^;UAS-dsRNAi ClC-c^CG1663^/UAS-dsRNAi ClC-c^CG1663^; ^+/+^ (VDRC_6465/GD)], eGFP tagging [*w*^−^;UAS-ClC-c::eGFP/UAS-ClC-c::eGFP; ^+/+^ (Burke Lab)] or with wildtype [*w*^*1118*^;^+/+^;^+/+^ (BDSC_3605)] for control comparisons. For UAS-dsRNAi ClC-c^CG1663^ the VDRC catalog #6465/GD was used instead of VDRC #106844/KK because the KK (phiC31) insertion occurs within a transcription factor, and the intracellular pH was found to be unstable (data not shown). Principal cells were selected over stellate cells on the basis of previous KD screenings by Cabrero and colleagues.^34^ All experiments using *Drosophila* were performed on female flies.

### Malpighian Tubule Staining

Malpighian tubules were dissected from *Drosophila* in Schneiders Medium and mounted on poly-L coated slides. Mounted tubules were fixed in 4% paraformaldehyde iPBS solution (121.5 mM NaCl, 20 mM KCl, 20 mM glucose, 8.6 mM HEPES, 10.2 mM NaHCO_3_, 4.5 mM NaH_2_PO_4_(H_2_O), pH 6.8, osmolarity 300±5 mOsm) at room temperature for 10 minutes and then washed, 4 times, in iPBS. The basolateral membrane was stained by Wheat Germ Agglutinin Alexa Fluor 647 Conjugate (WGA; Invitrogen, Thermo Fisher Scientific, Waltham, MA) diluted 1:100 in iPBS for 5 minutes and then washed, 4 times, in iPBS. Nuclei were stained with 4′,6-diamidino-2-phenylindole (DAPI; Thermo Fisher Scientific, Waltham, MA) diluted 1:10,000 in iPBS for 3 minutes and washed with iPBS before applying a coverslip with Dako fluorescence mounting medium (Agilent Technologies, Santa Clara, CA).

### *Drosophila* Crystallization Assays

Malpighian tubule crystal formation was induced *in vivo*, by a sodium oxalate (NaOx) diet, or *ex vivo*, by incubating tubules in medium containing NaOx. For *in vivo* assays, diets were prepared by mixing NaOx solution to melted standard diet to yield 20 mM solution and then allowing the diet to solidify at room temperature. *Drosophila* were transferred from vials containing standard diet to vials with 20 mM NaOx-enriched diet. After the designated time point, *Drosophila* were subjected to MT dissection in iPBS solution, and tubules were mounted on poly-L–coated slides in iPBS for polarized light microscopy of crystals. In *ex vivo* experiments, MTs from both control and ClC-c-KD flies were first dissected in Schneider's medium and mounted together on a poly-L–coated slide in iPBS. Images were collected by polarized light microscopy before and after incubation with 20 mM NaOx in iPBS for 1 hour.

### Secretion Assays

Malpighian tubule secretions were collected following the Ramsay Assay protocol.^39^ Briefly, MTs were dissected in Schneider's *Drosophila* medium and submerged through mineral oil into a 10 ul bath containing 1:1 Schneider's insect medium: modified secretion saline (94 mM NaCl, 16 mM KCl, 1.6 mM MgCl_2_, 1.6 mM CaCl_2_, 6.9 mM HEPES, 0.45 mM NaH_2_PO_4_(H_2_O), 1.0 mM NaHCO_3_, 20 mM glucose, pH 6.8), with 10 *μ*M of *Drosophila* kinin added as a diuretic. For protein secretion quantification, bovine serum albumin was added in the solution for a final concentration of 10 ug/ul. Protein from pooled secretions (4–6 tubules, 1 hour) was detected by Pierce Gold Assay and quantified by UV absorbance at 480 nM by Nanodrop ND-1000 (ThermoFischer Scientific, Waltham, MA). Ion chromatography was performed on individual ureter secretions collected for at least 50 minutes (mean±SEM; 92±5 minutes). For most tubules (37/51), a second droplet was collected, and mean values of the first and second droplet are reported. Droplets were diluted in 500 *µ*l ultrapure water using a Nanoject II (Drummond Scientific) and analyzed by cation chromatography. Chromatography was performed on a Dionex Integrion HPIC (ThermoFisher Scientific, Waltham, MA) system with AS-AP autosampler, IonPac CG12A column, CDRS 600 suppressor, and 25 *µ*l sample loop. Eluent was 25 mM methanesulfonic acid at 1 ml/min. Peak areas were converted to cation concentrations in secreted fluid on the basis of a 6-point standard curve (Dionex six cation-II standard, ThermoFisher Scientific, Waltham, MA), accounting for the dilution.

### Quantitative PCR

Expression of mRNA in MTs was assessed by quantitative PCR. MTs were dissected from flies in iPBS and placed in buffer RLT with 10% *β*-mercaptoethanol lysis buffer for RNA isolation. Once ≥50 tubules were pooled, RNA was isolated by Qiagen RNeasy Mini Kit (Qiagen Sciences Inc, Germantown, MD) with added DNase digestion step and eluted in 30 ul nuclease-free water. Total RNA was quantified by Nanodrop ND-1000. Then, cDNA was synthesized by Invitrogen Superscript III First Strand Synthesis (ThermoFisher Scientific, Waltham, MA) with 1:1 Oligo dT and Random Heximer primers. PCR products were verified by RT-PCR (30 cycles 98 melting/55C annealing) in an Eppendorf Mastercycler Pro S thermocycler (Eppendorf AG, Hamburg, Germany) and then quantified with a Roche Lightcycler 480 SYBR Green 1 on a Q-PCR (Roche Diagnostics, Indianapolis, IN) for 40 cycles.

### Microscopy

Images were collected by ZEISS Axio Observer 7 inverted wide-field or confocal microscopes equipped with Zen Blue 3.4 software (Carl Zeiss, Jena, Germany). Fluorescent images were collected using a Plan Neofluar 40×/0.6 air objective as Z-stacks and processed by orthogonal projections or Plan Apochromat 63×/1.4 oil objective with Airyscan Z-stacks processed in 3D. Fujii ImageJ was used for quantification of GFP fluorescence intensity of the whole tubule in 40× magnification with corrections made from background fluorescence. Tubule crystal images were collected by polarized light microscopy *via* a Plan Apochromat 10×/0.45 air observer with in-line light polarizers. Tubule images were collected in tiled fields and processed together as stitched. Crystal number and cross-sectional area were determined by Fuji ImageJ 3D Objects Counter. Total crystal area was calculated as the sum of crystal areas. All image thresholds were determined by control tissue images that were collected at the same time.

### Statistics

Statistical comparisons were compiled in GraphPad Prism 9.5.1 (GraphPad Software, Boston, MA). Electrophysiology traces were compared by using two-way ANOVA with Bonferroni comparisons or one-way ANOVA with Bonferroni comparisons when comparing groups at single voltages. Comparisons between knockdown or GFP-labeled and control *Drosophila* were made by t-tests analysis and paired when date of collection was identified as an interaction. Differences were considered significant when *P* < 0.05.

## Results

### Sequence Homology of ClC-c to CLCN-5

**Figure 1:**
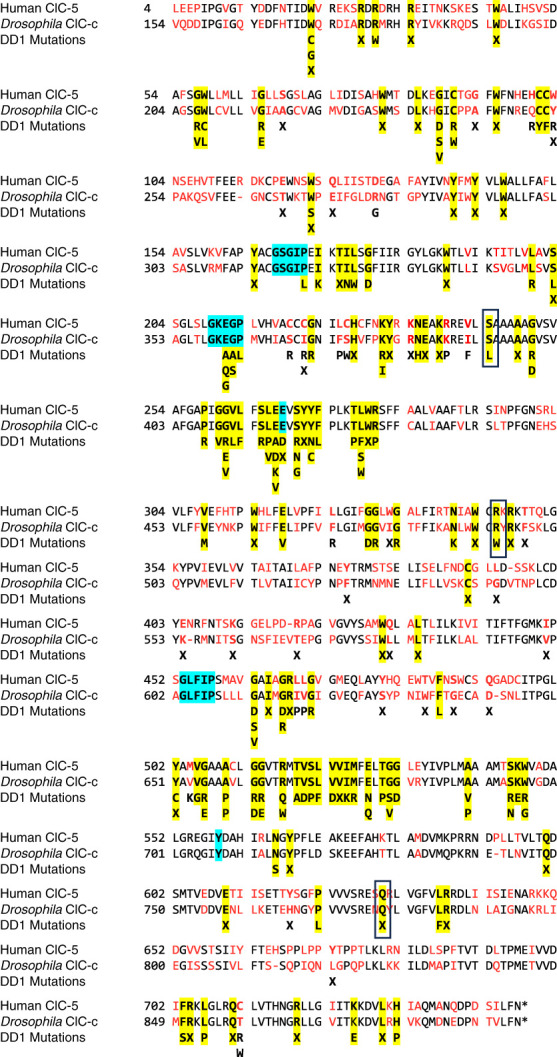
**Sequence alignments.** Segment of human ClC-5 (NP_000075.1, 746 a.a.; top rows) amino acid sequence stacked in alignment with *Drosophila* ClC-c (middle rows) and DD1 pathogenic variants (FBgn 0036466, 893 a.a.; bottom rows). Black lettering represents identical amino acids, whereas red represents different amino acids between human and *Drosophila*. Yellow highlighted amino acids represent locations of presently known DD1 point mutation sites.^6,37,43,44,54–59^ Cyan highlighted amino acids are involved in Cl^−^ selectivity and/or proton gating.^5,13,15,21,45^ Boxed regions depict mutations used for electrophysiological experiments.

The DIOPT Ortholog Prediction Tool (v9.1, flybase.org) shows that *Drosophila* ClC-c (FBgn 0036566) has greatest homology with CLCN3, CLCN4, and CLCN5 human sequences. *Drosophila* ClC-a and ClC-b also have homology with human CLCN sequences, but ClC-a is expressed in MT stellate cells and participates in fluid transport (Cabrero 2014), and ClC-b is not expressed in the MT. Both CLCN3 and CLCN4 have similar functions to CLCN5; however, these genes are associated with neural defects such as intellectual disability, epilepsy, and synaptic neural function more so than kidney dysfunction.^40,41^ Because DD1 affects the kidney, we have focused our investigation on ClC-c, which is highly expressed in the MT (Fly Cell Atlas scRNA-seq). The amino acid sequence of *Drosophila* ClC-c (FBgn0036566) aligns to human ClC-5 (NM_000084) with 62.9% sequence identity and 78.6% sequence similarity (Smith Waterman Alignment, (SnapGene version 6.2) and 52% sequence identity by NCBI Blast Global Amino Acid Alignment (https://blast.ncbi.nlm.nih.gov/Blast.cgi). The aligned sequences displayed in Figure 1 show common amino acids in black font and differences in red. Single-nucleotide polymorphisms that cause DD1 are known for 150 amino acids in ClC-5 and are depicted along with the alignment (HGMD, Aboudi 2023, *etc*.). *Drosophila* ClC-c has identical amino acids (yellow highlights) with 79 of the 93 DD1 sites (85%) associated with point mutations and 38 of 57 DD1 sites (67%) associated with only truncation. Key regions for ion selectivity (cyan highlight) in ClC-5, including GSGIP (167-171), GKEGP (209-213), GLFIP (453-457), and Y558^21^, were entirely conserved in ClC-c with GSGIP (316-320), GKEGP (358-362), GLFIP (603-607), and Y707, respectively. Proton gating (cyan highlight) of ClC-5 by E211 and E268 were also conserved in ClC-c at E360 and E417.^5,13,15,17^

### Cl^−^ Transport by ClC-c and pH Dependence

**Figure 2:**
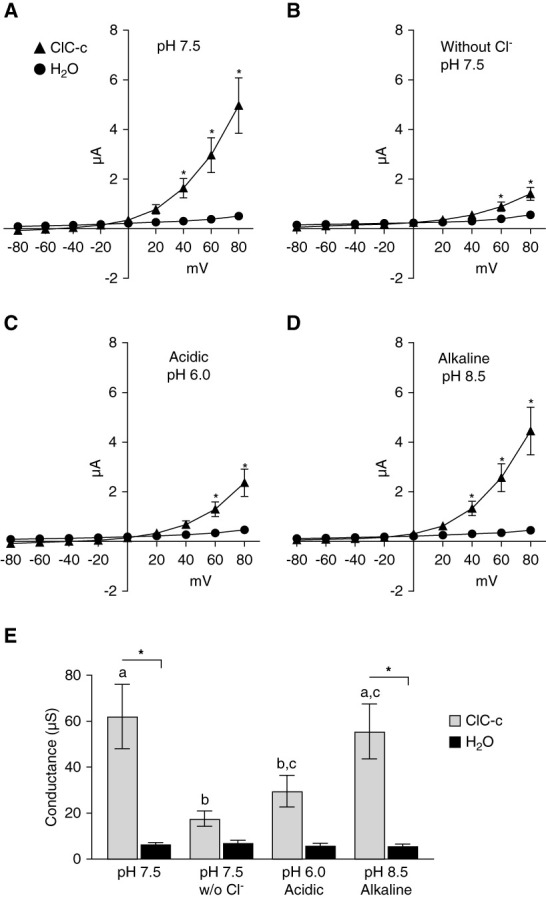
**ClC-c expressed in Xenopus oocytes is electrogenic, voltage-gated, and pH sensitive.** Voltage clamp experiments were conducted on oocytes injected with ClC-c cRNA or H_2_O perfused with ND96 solutions of pH 7.5 (A), pH 7.5 without Cl^−^ (B), pH 6.0 (C), and pH 8.5 (D). *Significantly different from respective H_2_O-injected oocytes or as indicated by two-way ANOVA analysis with Bonferroni post-test. The conductance observed +80 mV compared across solutions by one-way ANOVA. Error bars represent mean±SEM. (E) Conductance from ClC-c–injected oocytes with the same *α*-notation (a, b, c) are statistically similar (*P* ≥ 0.05). *Significantly different conductance from H_2_O-injected oocytes for the respective solution (*P* < 0.05).

In perfusion voltage clamp experiments, ClC-c–injected oocytes (*n*=8) generate strong outward rectifying currents that are not observed in H_2_O-injected controls (*n*=10; Figure 2). The currents generated by ClC-c were found to diverge significantly from controls at ≥ +40 mV (*P <* 0.001) under standard conditions (Figure 2A). When Cl^−^ in the solution is substituted with gluconate, the currents are greatly diminished and do not differ significant from controls until ≥ +60 mV. The effect of bath Cl^−^ suggests that the currents generated from ClC-c are related to the inward flow of Cl^−^ from the bath solution (Figure 2B). Because mammalian ClC-5 is recognized to exchange H^+^ for Cl^−^, we tested both acidic and alkaline solutions on these oocytes. Increasing extracellular [H^+^] (pH 6.0) impaired the current delaying the divergence from controls to ≥ +60 mV (Figure 2C). The alkaline solution of pH 8.5 had no apparent effect on the current with difference from controls remaining significant at ≥ +40 mV (Figure 2D). By comparing conductance at +80 mV, it is apparent that decreasing [Cl^−^] and increasing [H^+^] in the solution both impair the amplitude of the current while decreasing [H^+^] does not (Figure 2E). These data demonstrate that Cl^−^/H^+^ exchange occurs *via Drosophila* ClC-c.

### Homologous DD1 Mutations Affect Cl^−^ Transport

**Figure 3:**
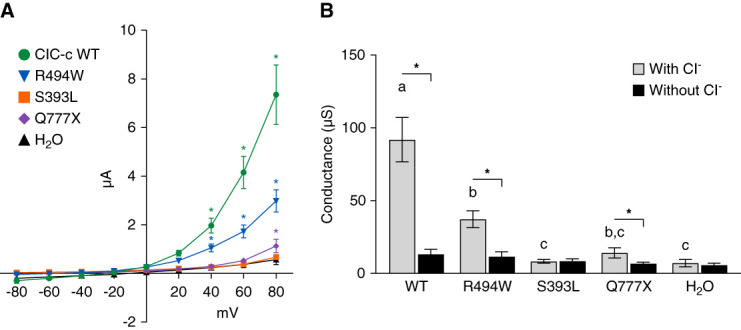
**Chloride transport by ClC-c with homologous DD1 mutations is impaired.** (A) Current responses to voltage clamp experiments for oocytes injected with wild-type ClC-c (WT), ClC-c with DD1 mutations (R494W, S393L, and Q777X), or H_2_O. *Significantly different from solution without Cl^−^’ by two-way ANOVA analysis with Bonferroni post-test (*P* < 0.05). (B) Conductances observed at 80 mV for each construct perfused by ND96 solutions with and without Cl^−^. Comparisons made by one-way ANOVA with Bonferroni comparisons. Error bars represent mean±SEM. Conductance from with Cl^−^’ solutions with the same *α* notation (a, b, c) are statistically similar (*P* ≥ 0.05). *Significantly different conductance between with Cl^−^’ and without Cl^−^’ solutions (*P* < 0.05).

Amino acids in ClC-c that correspond to select ClC-5 DD1 pathogenic variants, S244L, R345W, and Q629X,^36,37^ were mutated to create respective ClC-c homologs S393L, R494W, and Q777X (Figure 1, boxed sequences). In voltage clamp experiments, the wild-type ClC-c significantly increased current at ≥ +20 mV in standard solution compared with currents in solution without Cl^−^ (*P* < 0.001) and reached an amplitude of 7.4±1.2 *µ*A at +80 mV (*n*=9) (Figure 3). The outward rectifying current was observed with the R494W mutation (*P* < 0.001 at ≥60 mV compared with control); however, the amplitude of current at +80 mV (3.0±0.5 *µ*A, *n*=8) was approximately 41% of the WT (*P* < 0.0001). The S393L pathogenic variant (0.7±0.1 *µ*A, *n*=10) and Q777X-truncation (1.1±0.3 *µ*A, *n*=9) impaired function by 91% and 85%, respectively, and they did not differ from H_2_O-injected controls (0.6±0.2 *µ*A, *n*=9, *P* > 0.05).

### Voltage Gating

The outward rectifying currents generated by ClC-c indicate that a voltage-gated mechanism drives activity. To determine the gating voltage (V_g_), a segmented regression analysis was used that calculates the intersection point between two segments of a continuous line. The slopes of the current-voltage curve are indicated as conductance (G, in *µ*S). The line segment <V_g_ corresponds to the low-active state (G_L_), whereas the line segment >V_g_ is the active state (G_a_). To verify gating, the gated two-line segment model was compared with a nongated linear model. H_2_O-injected oocytes followed the linear model; therefore, V_g_ and G_a_ were not calculated. With ClC-c–injected oocytes, the V_g_ was 30±2 mV (95% confidence interval 26–33 mV) with no significant differences among treatments (*P*-values > 0.5, not shown; Table 1). No differences were observed among G_L_ of ClC-c–injected and/or H_2_O-injected controls. G_a_ for ClC-c decreased with Cl^−^ substitution and acidic solutions, but no effect was observed with the alkaline solution similar to Figure 2. All homologous DD1 mutations followed the line segment model, and V_g_ were similar; however, the G_a_ for mutations was significantly different from WT ClC-c. Furthermore, G_a_ for mutations was different from their respective G_L_, indicating that the mutations affect Cl^−^ transport rather than voltage gating.

**Table 1:** Gating and activity of ClC-c

Experiment	Gating Voltage V_g_ (mV)	Inactive Conductance G_L_ (*µ*S)	Active		Gating Effect *P*^b^
			Conductance G_a_ (*µ*S)	*P* ^a^	
Variable: Solution					
**ClC-c**					
pH 7.5	26.2±2.7	6.2±1.4	77.1±15.9	—	<0.0001^c^
pH 7.5, w/o Cl^−^	31.4±4.0	2.5±0.5	21.0±2.9	<0.0001^c^	<0.0001^c^
pH 6.0	27.0±6.1	3.5±0.5	41.6±10.2	0.02^c^	0.002^c^
pH 8.5	33.6±2.7	5.0±0.8	78.7±16.4	ns	<0.0001^c^
**H**_**2**_ **O**					
pH 7.5	—	2.3±0.3	—		
pH 7.5, w/o Cl^−^	—	2.1±0.4	—		
pH 6.0	—	2.1±0.4	—		
pH 8.5	—	1.9±0.3	—		
**Variable: Mutation**					
WT	30±3.3	10.3±1.9	141.3±30.4	—	<0.0001^c^
R494W	22.6±3.2	4.2±0.8	44.7±7.5	<0.0001^c^	<0.0001^c^
S393L	19.4±10.3	1.3±0.3	12.0±3.8	<0.0001^c^	0.01^c^
Q777X	30.8±8.1	2.1±0.3	28.6±10.4	<0.0001^c^	0.02^c^
H_2_O	—	4.5±1.7	—		

Data are displayed as mean±SEM.

a Comparisons with respective control condition by one-way ANOVA (ClC-c in pH 7.5 solution).

b Comparison between low active and active conductances to evaluate gating mechanism by t-test.

c Significant difference, not significant (ns).

### ClC-c Appears Along the Apical Membrane of the Malpighian Tubules

**Figure 4:**
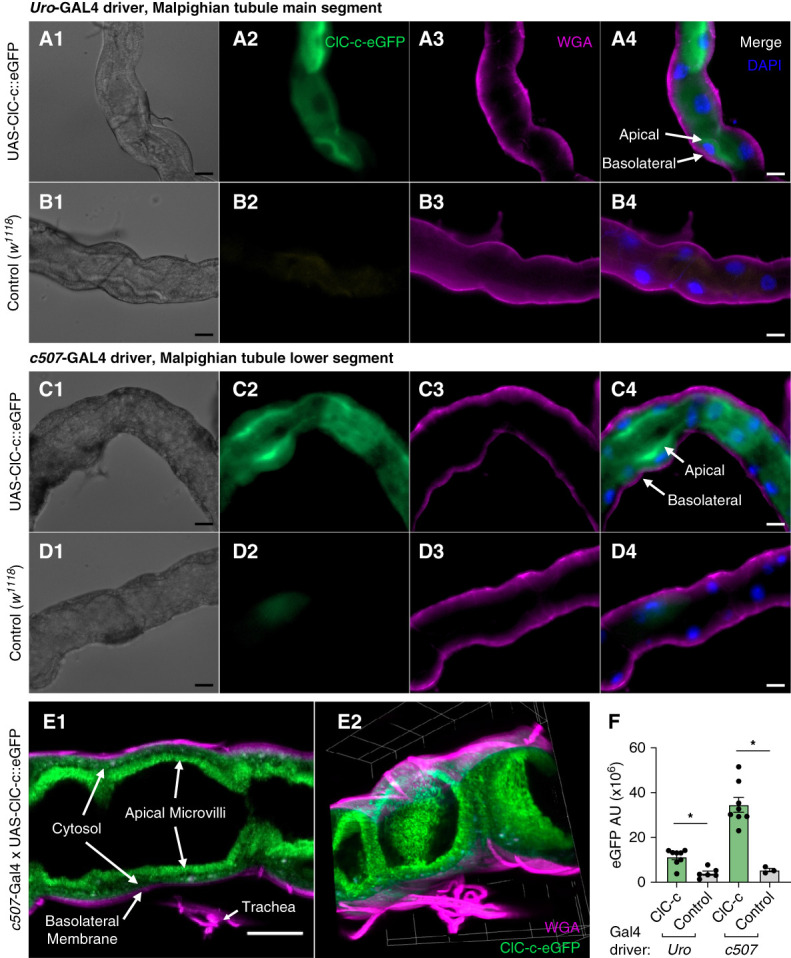
**ClC-c-GFP expressed in Malpighian tubules orient to the apical membrane and cytoplasm, but not the basolateral membrane.** Apical localization is evident with both *Uro*-GAL4 and c507-GAL4 principal cell drivers (A4, C4, and E), as indicated by labeled arrows. Images include MTs from female UAS-ClC-c::eGFP (rows A, C, E) or control (*w*^*1118*^, rows B, D) crossed with *Uro*-GAL4 (rows A, B) and *c507*-GAL4 (rows C, D, E) drivers 7–14 days after eclosure. Confocal images of *c507*-Gal4 x UAS-ClC-c::eGFP in €(E) with labels for apical microvilli, cytosol, basolateral membrane, and trachea and rendered in 3D (E2). Dissected tubules were fixed with 4% paraformaldehyde (A–E), the basolateral membrane was stained with wheat germ agglutinin [WGA, (A–D)3, E1, E2], and nuclei are stained with DAPI in merged images at 40× magnification [(A–D)4]. Scale bars=20 *µ*m. (F) Quantification of eGFP fluorescence (background corrected) from tubule traces in 40× images and analyzed by T-test comparisons with significant differences of *P < 0.001.

Expression of UAS-ClC-c::eGFP was driven selectively in tubules with GAL4 drivers, *Uro-*GAl4 (expressed in the main segment, principal cells) and *c507-*GAl4 (expressed in the lower tubule segment and ureter, principal cells). Wild-type controls were used to assign thresholds for fluorescent channel intensities above autofluorescence in each channel. The *Uro*-GAl4 driver resulted in intense ClC-c-eGFP concentrated in a few principal cells at the midpoint of the tubule with lower expression distributed throughout the main segment. The *c507* driver induced high expression in a distinct region of the tubule near the ureter. Regardless of driver, ClC-c localizes predominantly in the microvilli at the apical membrane of the MTs, with some GFP evident in the cytosol (Figure 4). No GFP was present at the basolateral membrane or in nuclei.

### ClC-c-KD in *D*. *melanogaster*

**Figure 5:**
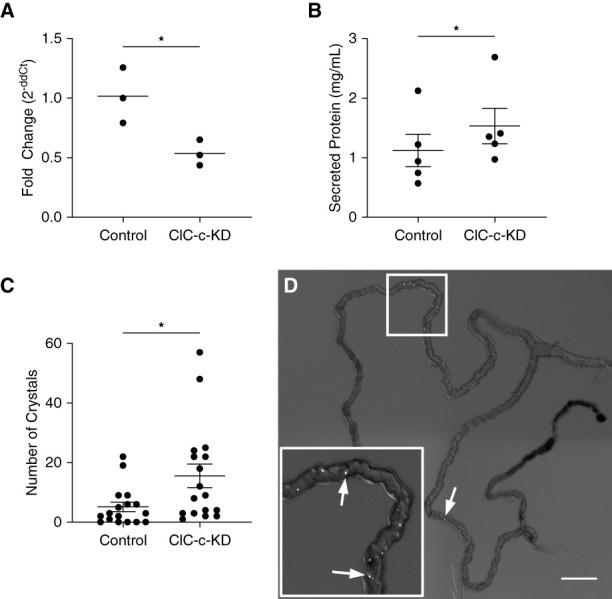
**Knockdown of ClC-c in *Drosophila* in the Malpighian tubules, 7 days after eclosure.** Knockdown of ClC-c was accomplished by crossing UAS-ClC-c-RNAi female flies with *Uro*-GAL4 male flies. Control crosses used female *w*^1118^ flies with *Uro-*GAL4 males. (A) qPCR expression of ClC-c mRNA in the tubules normalized to Rpl32 mRNA and relative to average of controls. (B) Concentration of protein secreted from the tubule ureter and compared by using the paired t-test. (C) Number of crystals in tubule pairs imaged by polarized light microscopy at 10× and quantified by ImageJ. *Significantly different by t-test, *P* < 0.05. Error bars represent mean±SEM. (D) Representative image of Clc-c-KD tubule pair with arrows depicting crystals. Scale bar=200 *μ*m.

UAS-ClC-c RNAi flies were crossed with the *Uro*-GAL4 driver to knockdown expression of ClC-c in MT principal cells. The resulting F1 generation had a 48% decrease in ClC-c mRNA expression in anterior MTs compared with controls 7 days after eclosure (*P =* 0.03, *n*=3 groups of approximately 50 pooled tubules; Figure 5A). On standard maintenance diet alone, tubule crystals were observed in all ClC-c KD flies, averaging 15±4 crystals per anterior MT pair (*n*=17), whereas crystals were observed in 71% of the control flies with fewer crystals than the KD flies (5±2 crystals per pair, *n*=17, *P* = 0.02; Figure 5C). Figure 5D illustrates birefringence of CaOx crystals observed in MT.

### Malpighian Tubule Secretions

*Ex vivo* MT secretions were collected under kinin diuretic stimulation. Protein secretion *via* ClC-c-KD MTs (1.53±0.30 mg/ml protein) was significantly greater than control (1.12±0.27 mg/ml protein, *P* = 0.038), when adjusting for date of collection by paired t-test (Figure 5B). Secretion rate and cations, including Na^+^, K^+^, NH_4_^+^, Ca^2+^, and Mg^2+^_,_ were measured by HPIC (Table 2). ClC-c-KD MTs had numerically lower secretion rate than controls, but this was not statistically different (*P* = 0.13). Only Ca^2+^ had a notable difference such that ClC-c-KD MT secretions had a higher concentration of Ca^2+^ (6.3±0.8 mM Ca^2+^) than controls (4.1±0.5 mM Ca^2+^, *P =* 0.018). Mg^2+^ was also greater for ClC-c-KD, but this trend was not significant (*P =* 0.082). Secretion of all other cations measured (Na^+^, K^+^, and NH_4_^+^) was not different between groups.

**Table 2:** Concentration of ions secreted from individual Malpighian tubules

Cation (mM)	Control (*n*=27)	ClC-c-KD (*n*=24)	*P*
Sodium, Na^+^	25.0±3.1	30.9±5.6	0.4
Potassium, K^+^	138.0±7.3	126.1±9.1	0.3
Ammonium, NH_4_^+^	7.32±0.74	8.00±0.90	0.6
Calcium, Ca^2+^	4.07±0.48	6.26±0.77	0.018^a^
Magnesium, Mg^2+^	3.73±0.30	4.66±0.52	0.082
Secretion rate (nl/min)	1.08±0.15	0.78±0.13	0.13

a Statistically significant by t-test comparison between control and ClC-c-KD, *P* < 0.05.

### Sodium Oxalate–Induced Crystallization in the Malpighian Tubules

**Figure 6:**
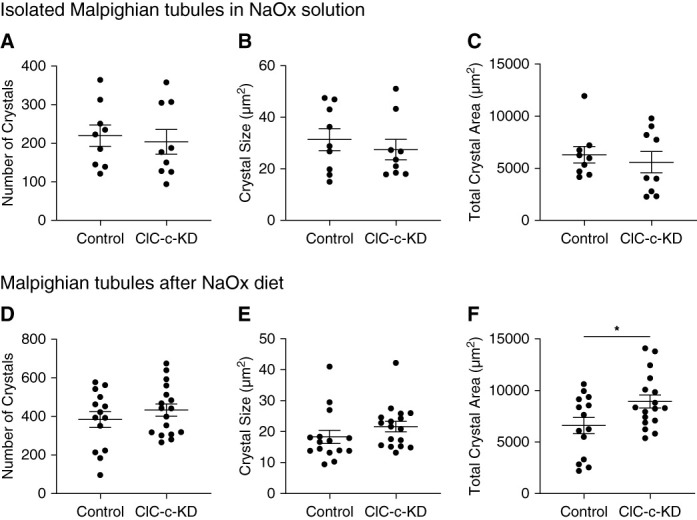
**Crystals in Malpighian tubules induced by sodium oxalate.** *Ex vivo* assessment of isolated MTs in 10 mM NaOx solution for 1 hour (A–C). Crystals from in MTs by 20 mM NaOx supplemented diet for 24 hours (D–F). Knockdown of ClC-c was accomplished by crossing UAS-ClC-c-RNAi female flies with *Uro*-GAL4 male flies. Control crosses used female *w*^1118^ flies with *Uro-*GAL4 males. Tubule pairs were imaged at 10× magnification by polarized light microscopy and quantified by ImageJ. Error bars depict mean±SEM. *Significantly different by t-test comparisons, *P* < 0.05.

ClC-c-KD flies had more crystals in the anterior MT than control flies; thus, KD of ClC-c may promote calcium oxalate (CaOx) precipitation and crystallization. Therefore, CaOx crystallization was induced both *ex vivo* in isolated tubules and *in vivo* by dietary supplementation. Tubules dissected from ClC-c-KD flies and submerged in 10 mM NaOx solution for 1 hour formed 204±32 crystals (*n*=9) and were similar to control flies (220±27 crystals, *n*=9, *P* = 0.7; Figure 6A). Both average surface area (27.5±4.0 *µ*m^2^, ClC-c KD versus 31.3±4.2 *µ*m^2^, control, *P* = 0.5) and total crystal area (5580±1020 *µ*m^2^ ClC-c-KD versus 6300±786 *µ*m^2^ control, *P* = 0.6) were similar between groups (Figure 6B, 6C). ClC-c-KD and control flies that were fed a diet of 20 mM NaOx for 1 day produced similar number of crystals (432.5±32 crystals, ClC-c-KD versus 383.8±41 crystals control; *P* = 0.3), with similar areas (21.65±1.7 *µ*m^2^, ClC-c-KD versus 18.3±2.1 *µ*m^2^, control; *P* = 0.2; Figure 6D, 6E). However, the total crystal area (sum of crystal areas per pair of tubules) was significantly greater in the ClC-c-KD MTs (8934±641 *µ*m^2^) than controls (6597±785 *µ*m^2^, *P* = 0.03, Figure 6F).

## Discussion

Hypercalciuria and Ca^2+^ dysregulation are prominent diagnostic criteria for DD1, but the relationship between this 2Cl^−^/H^+^ transporter and Ca^2+^ metabolism remains unclear. In this study, we evaluated *Drosophila* ClC-c as a human ClC-5 homolog to establish an alternative model for deciphering the Cl^−^ and Ca^2+^ connection. *Drosophila* models offer advantages which are unavailable or difficult to implement in mammalian models. Genetic manipulations are particularly useful as a variety of tools, such as RNAi knock-down and fluorescent ion sensors, are already developed, readily available, and can be expressed in specific tissues or cells. In addition, implementation of genetic constructs or breeding schemes can be accomplished rapidly (life cycle <2 weeks) and cost effectively (<$50 USD for a line and almost no daily costs), particularly when compared with mammalian models. To justify using the *Drosophila* model, we start by asking, how well does this *Drosophila* ClC-c compare with mammalian ClC-5?

*In silico*, human ClC-5 and *Drosophila* ClC-c sequences align with only modest homology, but key amino acids remain conserved and predict equivalent functions. In addition, both ClC-5 and ClC-c are highly expressed in their respective renal organs. The ion transport activities of human, rat, and mouse ClC-5, as well as for *E*. *coli* ClC-ec1, are characterized as eliciting strong outward rectifying currents that are gated at ≥ +20 mV.^6,13,20,36,37,42–44^ This electrogenic action of these transporters depends on Cl^−^ (or other halide ion) in the extracellular bath solution and is hindered by acidic pH.^5,13,20,36,37,42,45^ With *Drosophila* ClC-c, similar experiments unveiled the same outward rectifying current gated at +31 mV. Ion transport by *Drosophila* ClC-c is dependent on [Cl^−^] and decreased by acidic pH. The similarities in ion transport bolster the highly conserved role for this Cl^−^/H^+^ exchanger in the renal organ throughout evolution.

Dent disease type 1 is caused by sequence variants in CLCN5 that alter ClC-5 protein functions. The mutations S244L, G261E, G333R, and R345W; in-frame deletion 523delV; and Q629X truncation have all shown impaired Cl^−^ transport *in vitro*.^6,12,36,37,44^ Disease severity relates to the type of mutation such that patients with truncating mutations, such as Q629X, are prone to developing more severe phenotypes.^11^ Phenotypes associated with nontruncating mutations vary on the basis of the degree to which effective Cl^−^ transport is altered. Most of the nontruncating DD1 pathogenic variants obstruct ion transport; however, the R345W variant allows voltage gating, but impairs only the amplitude of currents generated. On investigation, Chang and colleagues found that the human R345W construct is entrapped in the endoplasmic reticulum and *cis*-Golgi without translocation to endosomes and the plasma membrane.^36,37^ Chloride transport was similarly assessed for *Drosophila* ClC-c with DD1 homologs: S393L, R494W, and Q777X. As expected, Cl^−^ transport, but not voltage gating, is affected by these mutations. Similar to the human R345W variant, the *Drosophila* R494W variant retained voltage gating, with a shift to +17.5±6.9 mV, but the amplitude of ion transport was decreased to 1/3 of its activity. One can reason that this *Drosophila* R494W mutation may also cause impaired trafficking in ClC-c and warrants additional investigations related to subcellular localization. Ultimately, homologous DD1 mutations in ClC-c do hinder ion transport and justify additional KD strategies to model DD1.

Identifying the cellular expression and localization of an epithelial transporter leads to understanding its physiological role in an organism. In the mammalian kidney, ClC-5 is expressed in proximal tubule and *α*-intercalated cells where solute reabsorption occurs. Subcellularly, the human transporter appears along the apical membrane and among endosomes where it co-localizes with vacuolar H^+^ ATPases (*i*.*e*., V-ATPases).^1–4,12,22,46^ *Drosophila* and other insects use MTs as their analogous renal organ to maintain osmotic homeostasis of the hemolymph with principal cells providing cation secretion and stellate cells maintaining osmoregulation.^30,31,34,47^ Principal cells also use V-ATPase in a manner reminiscent of the proximal tubule and *α*-intercalated cells in mammals.^48,49^ Transcriptomics have shown that ClC-c is highly expressed in the adult MTs, and work by Cabrero and colleagues noted that ClC-c is more prominently expressed in principal cells than stellate cells.^34,50,51^ Here, GFP-labeled ClC-c expressed in principal cells localized to the apical microvilli and in the cytosolic regions along the apical membrane, suggesting that, like mammalian ClC-5, *Drosophila* ClC-c likely incorporates within endosomes. The similarities in localization suggest that the physiological comparisons between mammalian ClC-5 and *Drosophila* ClC-c are compatible and justify in-depth assessments of the role of ClC-c in endosomal acidification and trafficking for future studies that exploit the variety of genetic tools of the *Drosophila* model.

Knockdown of ClC-c in *Drosophila* adult MTs reveals a striking resemblance to the phenotype described for patients with DD1. DD1 is recognized to be a rare kidney stone disease with the two most prevalent characteristics being low molecular weight proteinuria and hypercalciuria. With a modest 48% decrease in mRNA expression, the ClC-c-KD flies exhibited increases in *both* Ca^2+^ and protein secretion. Not only did the KD MTs secrete more protein and Ca^2+^, but they also had more crystals form spontaneously than control flies. In dissected MTs, ClC-c KD did not manifest as more CaOx crystal formation. However, when fed with a high sodium oxalate diet, total crystal area was greater with the KD although crystal number and size were not different. The differences in crystal formation between diet and isolated MTs suggest that the cause of crystal formation has a systemic aspect and is not resulting from mishandling of oxalates in principal cells. Because KD flies do have more spontaneous crystals, we should consider that the composition of these crystals may contain complex precipitates besides CaOx. Mixed composition crystals resemble the stones associated with DD1, which are composed of both CaOx and calcium phosphate.^7,8,52,53^ Although CaOx crystals are relatively straightforward to assess using polarized light and birefringence, Ca-phosphate crystals require histologic stains for both Ca and phosphate and are not observed with birefringence. These staining requirements do not allow for coincident staining and also do not allow investigators to follow crystal formation as it is occurring. Nevertheless, the fly model reported here seems to recapitulate disease characteristics of human DD1 and may be an alluring complement to previous rodent models.

The model that we have described has inherent limitations and may limit a direct translation to human or DD1. The genetic contribution is an important limitation to consider. DD1 is X-linked and affects males more aggressively than female carriers. In *Drosophila*, ClC-c is on chromosome 2 and not sex-linked. Thus, male and female flies are assumed to be affected similarly. As for phylogenic differences, *Drosophila* do not require Ca^2+^ for bone formation and are not susceptible to extra-renal skeletal maladies, such as rickets or fracture. Female flies, however, require additional Ca^2+^ metabolism for egg production and only female flies were used in assessments and analytics.

In conclusion, we compared the functional attributes of *Drosophila* ClC-c with mammalian ClC-5 and have introduced the ClC-c-KD fly as an avatar to model DD1. This model recapitulates the two most prevalent features of DD1, with increased secretions of both protein and Ca^2+^, as well as produces notable increases in spontaneous tubule crystal formations. Future investigations with this model will offer extensive tools for unraveling the relationship between this Cl^−^ transporter and increased Ca^2+^ secretions and studying endosomal pH and Ca^2+^ transport, all of which are critical for improving management of patients with DD1.

## Supplementary Material

## Data Availability

All data are included in the manuscript and/or supporting information.

## Supplemental Material

Supplemental Table 1. Primer sequences for RT-PCR, subcloning, and mutagenesis.
