# Supplementary material for: Drosophila ClC-c Is a Homolog of Human CLC-5 and a New Model for Dent Disease Type 1
Source: Kidney360. 2024 Jan 18;5(3):414–26. doi: 10.34067/KID.0000000000000352 (PMC11000744; doi:10.34067/KID.0000000000000352)
Supplement: Supplementary file 1 [file kidney360-5-414-s001.pdf]

Supplemental Material

Drosophila ClC-c is a homolog of human CLC-5 and a new model for Dent disease type 1.

Carmen J Reynolds, Christopher M. Gillen, Richard Burke, Yula Tsering, Emi Loucks, Sebastian Judd-Mole, Julian A T Dow, Michael F Romero

Table of Contents

Supplemental Table 1. Primer sequences for RT-PCR, subcloning and mutagenesis.

Supplemental Table 1: Primer sequences for RT-PCR, subcloning and mutagenesis.

Target	Forward (5'-3')	Reverse (5'-3')
RT-PCR		
ClC-c	TCCACCCGCCTTTTGGTTTA*	GCCCACAGCACATACCAGAT
ClC-c (C-term)	GTGCGGTATATTGTGCCCCTGATG*	
RPL32	ATGCTAAGCTGTCGCACAAATG	GTTCGATCCGTAACCGATGT
Subcloning		
ClC-c for pGEMHE vector	CGGGGATCCGAATTC-TGATCGCAGGATGGAGAAGTTTCCGCT	AACCAGATCAAGCTT-TCAGTTGAAGAGCACCGTATTGGGA
ClC-c for pUAST-attB: GFP vector	CGGGTACC ATGATTGACATCACACCC	GCTCTAGA GTTGAAGAGCACCGTATT
Mutagenesis		
ClC-c S393L	GATTCTTTTGGCAGCAGCAGCCGCAGG	GCTGCCAAAAGAATCTCACGCTTCTTGGCC
ClC-c R494W	GTGGTGCTGGTACAGGAAGTTCAGCAAGCTCGG	CTGTACCAGCACCACCACAAATTGGCC
ClC-c Q777x	GGGAGAATTAATATTTAGTGGGCTTTGTATTGCG	AATATTAATTCTCCCGCGACACCAC

Primers synthesized by Integrated DNA Technologies, Inc (Coralville, IA).

*RT-PCR Primers used for mutagenesis sequencing.
